# Development and Validation of a Clinical Symptom-based Scoring System for Diagnostic Evaluation of COVID-19 Patients Presenting to Outpatient Department in a Pandemic Situation

**DOI:** 10.7759/cureus.13681

**Published:** 2021-03-03

**Authors:** Aakashneel Bhattacharya, Piyush Ranjan, Arvind Kumar, Megha Brijwal, Ravindra M Pandey, Niranjan Mahishi, Upendra Baitha, Shivam Pandey, Ankit Mittal, Naveet Wig

**Affiliations:** 1 Infectious Diseases, All India Institute of Medical Sciences, New Delhi, IND; 2 Medicine, All India Institute of Medical Sciences, New Delhi, IND; 3 Microbiology, All India Institute of Medical Sciences, New Delhi, IND; 4 Biostatistics, All India Institute of Medical Sciences, New Delhi, IND

**Keywords:** covid-19, score, screening, validation, diagnosis

## Abstract

Background

Preventive strategies in the form of early identification and isolation of patients are the cornerstones in the control of COVID-19 pandemic. We have conducted this study to develop a clinical symptom-based scoring system (CSBSS) for the diagnostic evaluation of COVID-19.

Methods

In this study, 378 patients presenting to screening outpatient clinic with clinical suspicion of COVID-19 were evaluated for various clinical symptoms. Statistical associations between presenting symptoms and reverse transcription-polymerase chain reaction (RT-PCR) results were analysed to select statistically significant clinical symptoms to design a scoring formula. CSBSS was developed by evaluating clinical symptoms in 70% of the total patients. The cut-off score of the CSBSS was determined from ROC (receiver operating characteristics) curve analysis to obtain a cut-off for optimum sensitivity and specificity. Subsequently, developed CSBSS was validated in the external validation dataset comprising 30% of patients.

Results

Clinical symptoms like fever >100^0^F, myalgia, headache, cough and loss of smell had significant association with RT-PCR result. The adjusted odds ratios (95% confidence interval [CI]) for loss of smell, fever >100°F, headache, cough and myalgia were 5.00 (1.78-13.99), 2.05 (1.36-3.07), 1.31 (0.67-2.59), 1.26 (0.70-2.26) and 1.18 (0.50-2.78), respectively. The ROC curve and area under the curve of development and validation datasets were similar.

Conclusion

The presence of fever >100°F and loss of smell among suspected patients are important clinical predictors for the diagnosis of COVID-19. This newly developed CSBSS is a valid screening tool that can be useful in the diagnostic evaluation of patients with suspected COVID-19. This can be used for the risk stratification of the suspected patients before their RT-PCR results are generated.

## Introduction

The ongoing COVID-19 pandemic has already taken a great toll on human lives as well as on healthcare facilities all around the globe [1,2]. The diagnostic facilities are already overwhelmed by ever-increasing number of samples being tested for SARS CoV-2. In spite of the fact that all the countries are slowly adopting vaccination programmes based on their existing resources, we need a substantial time period to achieve herd immunity against this virus [3]. Preventive measures in the form of social distancing and the use of face masks play a pivotal role to break the chain of transmission of the virus [4-6]. But early detection and isolation of cases are still the mainstays of controlling the spread of this pandemic.

RT-PCR is the cornerstone of the diagnosis of SARS CoV-2 infection. The turn-around time for this molecular test varies on the basis of the testing platform used which ranges from a few hours to two days, based on the healthcare resources of that region [7]. We have developed a scoring system to determine the probability of a person being infected with SARS CoV-2, based on the clinical presentations. This is a simple and valid tool that helps to predict the laboratory results while the test reports are awaited.

Clinical symptom-based scoring systems (CSBSS) have been important in the early diagnosis and prognostic evaluation in different disease conditions. One of such scoring systems was developed during the influenza A (H1N1pdm 09) pandemic [8]. Attempts have been made to design a scoring system for the diagnosis of COVID-19 using both clinical and laboratory parameters [9]. In our study, we have evaluated the clinical symptoms among the patients with suspected COVID-19 presenting to the screening outpatient clinic of our healthcare facility to develop and validate a clinical symptom-based scoring system for the diagnostic evaluation of COVID-19.

## Materials and methods

We performed a single-centre, cross-sectional study among 378 adults who were tested for severe acute respiratory syndrome coronavirus-2 (SARS CoV-2) in an outpatient clinic at a tertiary care hospital in New Delhi, India, between June 17 to July 1, 2020. From 1066 suspected patients who were tested during this period, 384 patients were enrolled in the study based on the availability of informed consent and successful telephonic communication. Six patients were excluded from the study due to the non-availability of their test results due to pre-analytical issues. We collected socio-demographic and clinical data from the patients and their test reports were accessed from the hospital information system. To minimise bias, telephonic conversations were performed prior to the generation of test reports. The study protocol was approved by the Institute Ethics Committee, All India Institute of Medical Sciences, New Delhi and appropriate consent was obtained from the participants before their enrolment.

Participants were tested after a thorough clinical evaluation. Indications for SARS CoV-2 RT-PCR were based on the testing advisory developed by the Indian Council of Medical Research (ICMR), Version 5, dated May 18, 2020 [10]. One nasal swab and one throat swab were collected from each patient. AriaMx Real-time PCR System (Agilent Technologies, Santa Clara, CA) was used for molecular testing using commercially available real-time polymerase chain reaction (PCR) kits targeting E gene, N gene, ORF gene and RdRP gene. The combinations of gene targets used varied on the basis of different kit manufacturers. These two swabs were transported to the testing laboratory in a single vial of the viral transport medium maintaining the cold chain. The patients were interviewed telephonically and data was recorded in a pre-designed proforma. Data were collected after patients underwent testing and before their test results were generated.

Development and validation of the clinical symptom-based scoring system

The clinical symptom-based scoring system (CSBSS) was developed by a standard methodology. The patients (n=378) were divided into two datasets by a random selection process. Of these, 70% (n=265) of them were randomly selected as the development dataset while the remaining (n=113) were designated as the validation dataset (n=113). The development dataset (n=265) was used to develop CSBSS using clinical variables. Developed CSBSS was validated on the validation dataset (n=113), external to the development dataset.

Statistical analysis

Association between the clinical symptoms and RT-PCR results were assessed using Pearson’s chi-square test. For the development dataset, unadjusted and adjusted odds ratios were calculated to find out the strength of association between the presence of clinical symptoms and RT-PCR positivity. For log odds as an outcome variable, regression coefficients were computed for each clinical symptom. All the regression coefficients were divided by the lowest coefficient to obtain a score for each clinical symptom. Each score was multiplied by 10 so as to get the lowest score as 10. Using derived individual score for the clinical symptoms, a total score was obtained for every subject in the development dataset. Receiver operating characteristics (ROC) curve analysis was used to determine the appropriate cut-off for the total score. Sensitivity, specificity, positive and negative predictive values for the selected cut-off were determined. For validation of the CSBSS, individual scores for the clinical symptoms were used to obtain the total score for each candidate in the validation dataset, and the sensitivity, specificity, positive and negative predictive values for the obtained cut-off were compared with the development dataset. Stata 15.0 statistical software (StataCorp, College Station, TX) was used for data analysis.

## Results

Demographic and clinical data

We enrolled 379 adults who were tested for SARS CoV-2 by real-time RT-PCR between June 17 and July 1, 2020 in an outpatient screening clinic. This clinic caters to the staff and healthcare workers as well as their family members who are under the health scheme provided by the same hospital. The mean age of the participants was 35.6 ± 11.5 (SD) years, 246 (65.1%) were males and 132 (34.9%) were females.

COVID-19 RT-PCR was positive in 125 (33.1%) patients. The distribution of the clinical symptoms in the RT-PCR positive and negative patients in the development and validation dataset was similar to that of the total population (Table 1). Fever (60%) was found to be the most prevalent symptom in COVID-19 patients; followed by cough (44.8%), sore throat (42.4%), headache (28%), myalgia (21.6%) and breathlessness (12.8%). Gastrointestinal symptoms were present in 26 (20.8%) patients; the incidence of nausea, vomiting and diarrhoea were 8.8%, 2.4% and 9.6% respectively.Additionally, 28 (22.4%) patients complained of loss of smell while they were interviewed. We observed significant associations between a positive RT-PCR result with fever of >100°F (p <0.001), cough (p = 0.013), headache (p = 0.016), myalgia (p = 0.003) and loss of smell (p <0.001).

**Table 1:** Association of clinical symptoms with RT-PCR results in the derivation dataset, validation dataset and the total population

Clinical Symptoms	Development dataset (70%) n = 265			Validation dataset (30%) n = 113			Total population (100%) n = 378		
	RT-PCR			RT-PCR			RT-PCR		
	Positive, n = 83 (31.3 %)	Negative, n=182 (68.7%)	p-value	Positive, n=42 (37.2%)	Negative, n=71 (62.8%)	p value	Positive, n=125 (33.1%)	Negative, n=253 (66.9%)	p-value
Body temperature			<0.001			0.01			<0.001
< 100°F	35 (42.2)	107 (58.8)		24 (57.1)	47 (66.2)		34 (27.2)	19 (7.5)	
> 100°F	48 (57.8)	75 (41.2)		18 (42.9)	24 (33.8)		91 (72.8)	234 (92.5)	
Sore throat	44 (53.0)	118 (64.8)	0.06	14 (33.3)	19 (26.8)	0.4	53 (42.4)	83 (32.8)	0.06
Cough	39 (47.0)	51 (28.0)	0.002	17 (40.5)	15 (21.1)	0.02	56 (44.8)	66 (26.1)	<0.001
Headache	23 (27.7)	31 (17.0)	0.05	12 (28.6)	11 (15.5)	0.09	35 (28.0)	42 (16.6)	0.01
Myalgia	18 (21.7)	20 (11.0)	0.02	9 (21.4)	11 (15.5)	0.4	27 (21.6)	31 (12.3)	0.01
Breathlessness	12 (14.5)	19 (10.4)	0.3	4 (9.5)	4 (5.6)	0.4	16 (12.8)	23 (9.1)	0.2
Nausea	8 (9.6)	13 (7.1)	0.4	3 (7.1)	2 (2.8)	0.2	11 (8.8)	15 (5.9)	0.2
Vomiting	3 (3.6)	7 (3.8)	0.9	0 (0)	1 (1.4)	0.4	3 (2.4)	8 (3.2)	0.6
Diarrhoea	6 (7.2)	9 (5.0)	0.4	6 (14.3)	4 (5.6)	0.1	12 (9.6)	13 (5.1)	0.1
Loss of smell	19 (22.9)	6 (3.3)	<0.001	9 (21.4)	4 (5.6)	0.01	28 (22.4)	243 (96.1)	<0.001

Development of CSBSS

The development dataset (n=265) was used to develop the clinical symptom-based scoring system using the five selected clinical symptoms viz., fever >100°F, cough, headache, myalgia and loss of smell. Multivariable logistic regression analysis with these variables yielded specific scores against each clinical symptom from their odds ratios and regression coefficients (Table 2). These were used to formulate the following scoring formula:

Clinical symptom-based score = (41.7 x Fever >100°F) + (13.5 x Cough) + (15.8 x Headache) + (10 x Myalgia) + (94.7 x Loss of smell).

Values for the presence or absence of a symptom will be put into the scoring formula as 1 or 0, respectively. A score of > 41.7 is considered as COVID-19 positive (Table 2). 

**Table 2:** Multivariable logistic regression analysis showing odds ratios and scores assigned to different variables in the development dataset (n = 265) * Clinical score for each clinical symptom was obtained by dividing each of the coefficients by the smallest coefficient, i.e. 0.173 and multiplying by 10 CI: confidence interval

Variables	Unadjusted Odds Ratio (95% CI)	Adjusted Odds Ratio (95% CI)	Regression Coefficient (95% CI)	Clinical Score^*^
Temperature > 100^0^F	2.77 (1.65-4.64)	2.05 (1.36-3.07)	0.719 [0.31-1.12]	41.76
Cough	1.93 (1.14-3.24)	1.26 (0.70-2.26)	0.233 [-0.35-0.81]	13.52
Headache	2.07 (1.13-3.77)	1.31 (0.67-2.59)	0.276 [-0.39-0.95]	15.88
Myalgia	2.87 (1.40-5.89)	1.18 (0.50-2.78)	0.173 [0.67-1.02]	10.00
Loss of smell	7.14 (2.75-18.5)	5.00 (1.78-13.99)	1.60 [0.58-2.63]	94.70

**Figure 1:**
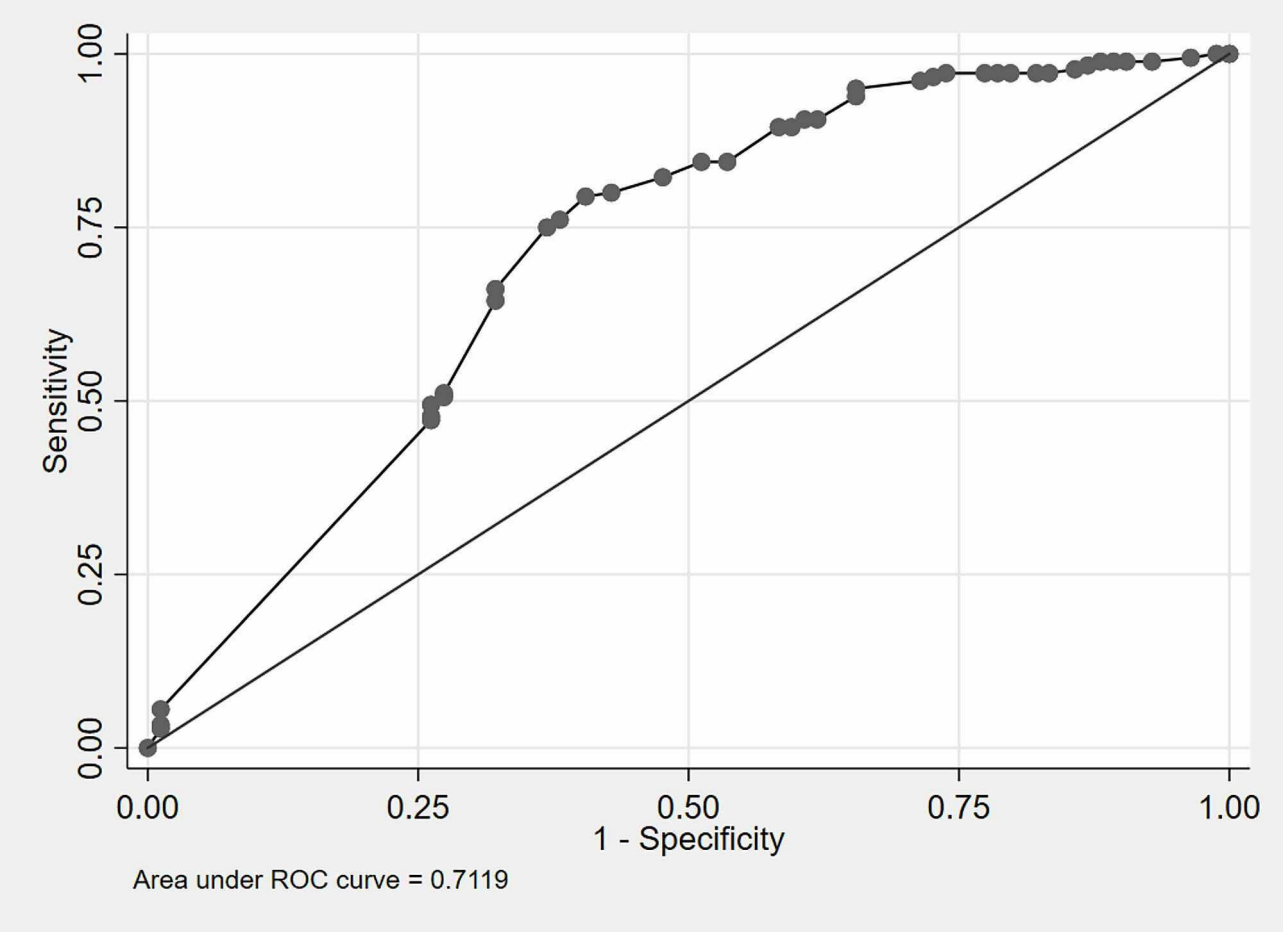
ROC Curve for development dataset Derivation dataset (70%), n=265 ROC: receiver operating characteristics

The sensitivity and specificity of this scoring system at different cut-off points are mentioned in Table 3. The ROC curve derived from the development dataset is shown in Figure 1. The cut-off score for this CSBSS was set at 41.7 to obtain optimum sensitivity and specificity values by the ROC curve generated from the development dataset. The area under the ROC curve (AUC-ROC) on the development dataset was 0.71 (95% CI: 0.61-0.80) (Figure 1), which demonstrated good discriminatory power. Sensitivity, specificity, positive and negative predictive values of the scoring system were 64.6% (95% confidence interval [CI]: 54.2-74.1), 62.1% (95% CI: 54.4-69.5), 49.2% (95% CI: 40.2-58.3) and 75.5% (95% CI: 67.5-82.4), respectively (Table 4).

**Table 3:** Sensitivity and specificity at different cut-off points of CSBSS CSBSS: clinical symptom-based scoring system

Cut-off points	Sensitivity	Specificity
10	75%	42.6%
15.8	68.7%	56.8%
41.7	64.6%	62.1%
51.7	56.2%	73.3%
57.6	51%	82.2%

**Table 4:** Comparison of CSBSS characteristics for development and validation datasets (at cut-off score 41.7) CSBSS: clinical symptom-based scoring system

Characteristics	Development dataset (70%) (95% CI)	Validation dataset (30%) (95% CI)
% Sensitivity (95% CI)	64.6 (54.2-74.1)	64.7 (46.5-80.3)
% Specificity (95% CI)	62.1 (54.4-69.5)	58.6 (46.2-70.2)
% Positive Predictive Value (95% CI)	49.2 (40.2-58.3)	43.1 (29.3-57.8)
% Negative Predictive Value (95% CI)	75.5 (67.5-82.4)	77.4 (63.8-87.7)
AUC (95% CI)	0.71 (0.61-0.81)	0.69 (0.62-0..76)

Validation of the scoring system

**Figure 2:**
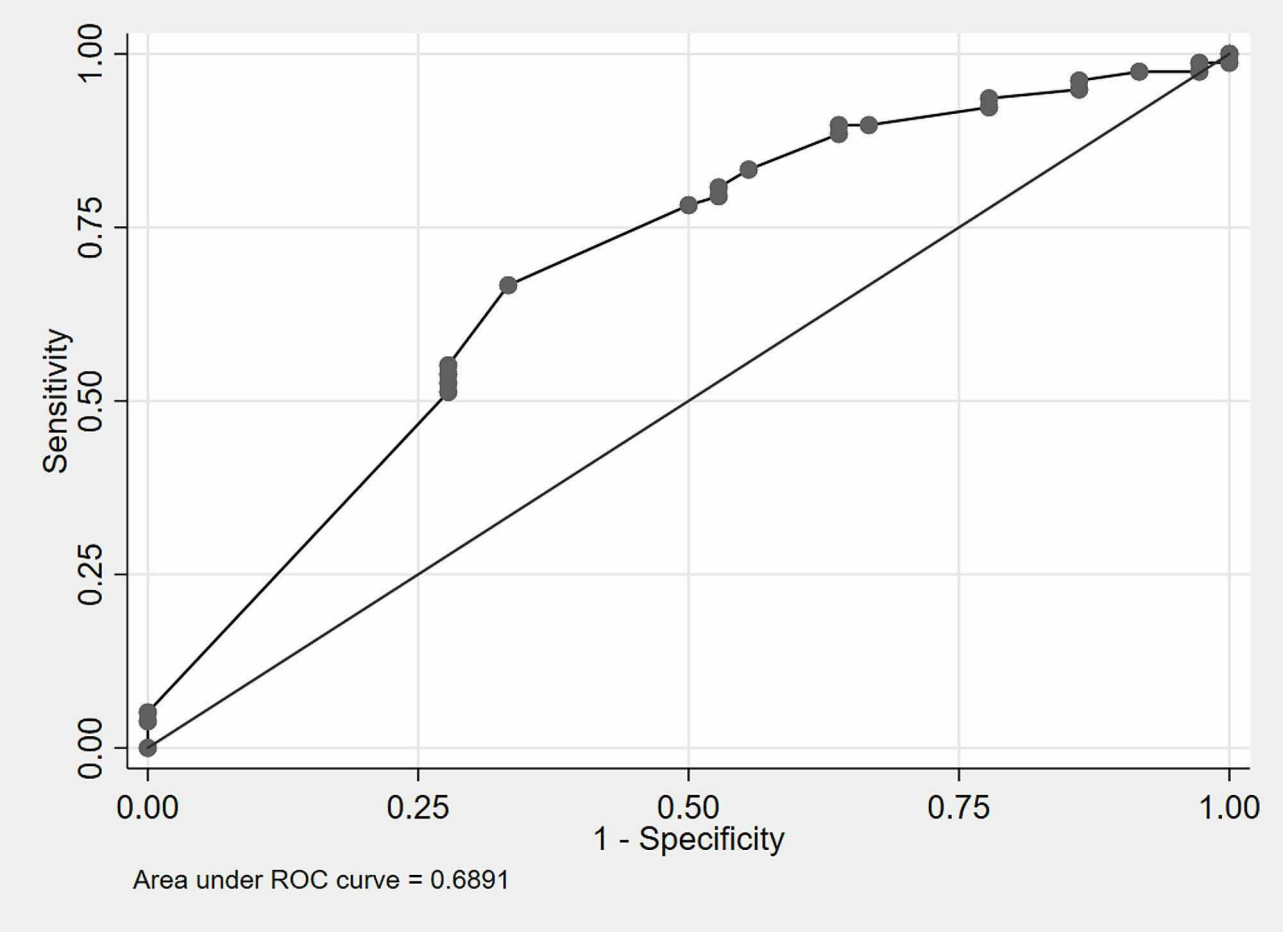
ROC Curve for validation dataset Validation dataset (30%), n=113 ROC: receiver operating characteristics

Validation of the newly developed CSBSS was performed on the validation dataset comprising 113 patients. It showed an AUC-ROC of 0.69 (95% CI: 0.62-0.76) (Figure 2), which is closely comparable with the AUC on the development dataset (Figure 1). The sensitivity (64.7%), specificity (58.6%), positive (43.1%) and negative predictive values (77.4%) for the validation dataset were similar when compared with the development dataset (Table 4).

## Discussion

We intended to develop a scoring system for diagnostic evaluation of COVID-19 based on presenting clinical symptoms. A similar scoring system was developed by Borghetti et al. which is based on both clinical and laboratory parameters [9]. Our clinical symptom-based scorings system for COVID-19 is the first of its kind as it is based upon the clinical symptoms only. The uniqueness of this study is that the diagnostic scoring formula will help in readily identifying and isolating the individuals who have a high probability of having the disease. The process of prioritizing suspects with a score above the cut-off (>41.7) requires only a mathematical calculation and can be performed in any healthcare or community setting. Assessment of the presence or absence of the five clinical symptoms (fever >100°F, myalgia, headache, cough and loss of smell) used in the formula can be performed even by personnel not involved with healthcare facilities.

We analyzed the statistical associations between the clinical symptoms with the RT-PCR results. The study was performed in an out-patient clinic in a tertiary care hospital and the study population was heterogeneous. All of the 378 participants were either symptomatic having an influenza-like illness (ILI) or asymptomatic persons with a history of direct or high-risk contact with a laboratory-confirmed COVID-19 patient. We could include mild to moderate disease patients (using the NIH/WHO definitions) only, as they were enrolled from a walk-in clinic.

Among 125 participants who were tested positive, 58 (46.4%) of them were symptomatic and 67 (53.6%) were asymptomatic at the time of the testing. Test candidates who did not have any symptoms, comprised of individuals who were direct or high-risk contacts of a laboratory proven COVID-19 case. Testing of the asymptomatic contacts was performed between five to 10 days from the date of contact, as per the Indian national COVID-19 testing strategy. These asymptomatic patients were not followed up in this study to determine whether they developed symptoms later on or not. Fever was the most predominant symptom among the positive patients and only 12.8% complained of shortness of breath as all of them were walk-in patients. The frequency of the clinical symptoms in our population was similar to a large study conducted among 1,420 mild to moderate symptomatic COVID-19 patients from 18 hospitals in five European countries [11].

The presence of fever of >100°F was positively associated (odds 2.05) with a positive swab test result, which is probably being reported by us for the first time from this study (Table 2). A very high degree of association (odds 5.00) was also found between anosmia and COVID-19 positivity which has already been described in several studies [12]. Besides, we had good associations of myalgia, headache, and cough with COVID-19 positivity (odds ratios 1.18, 1.31 and 1.26, respectively). Gastrointestinal symptoms did not have any significant association with the disease which is consistent with other studies describing them as atypical symptoms of COVID-19 [13].

The developed CSBSS consists of five clinical symptoms namely, fever >100°F, cough, myalgia, headache, and loss of smell. The derived formula for the scoring system was found to be internally valid. In the current pandemic situation, a diagnostic scoring system based on the clinical symptoms would be beneficial in predicting the probability of a positive test result, which can be performed at the first contact with suspect patients. This system will have a potential role in isolating high-risk subjects in areas where the turn-around time for the RT-PCR result is considerably longer. Thus, it will be useful to control this pandemic in countries or areas where healthcare resources are already exhausted or limited.

The cut-off score was derived by analyzing the ROC curve and computing a set of values of sensitivity and specificity at different cut-offs. We decided to fix the cut-off score at 41.7 to obtain optimum sensitivity and specificity of 64.6% and 62.1%, respectively. As the sensitivity of this system is not very high, we can’t use this as a screening test in isolation. By the individual scores derived from the patients, we can prioritize the high-risk candidates to find out those who need the confirmatory molecular tests at the earliest. Moreover, we can objectively classify a suspect patient as a “probable case” which will help in early and effective isolation of such patients. As the use of this scoring system will lead to some false negative results, individuals assessed by this scoring system should continue to adhere to the standard infection prevention practices (social distancing, use of face masks etc.) till the molecular test reports are available.

If the cut-off is set to a lower value, this sensitivity of the scoring system increases but it comes at the cost of a lower specificity (higher false-positive rate). We already have rapid point of care screening tests for COVID-19 in the form of antigen detection tests which offer good specificity values. The major drawback of these tests is their low sensitivity [14]. Rapid antigen tests are usually used to decrease the turn around time of the results or where RT-PCR facilities are not available. Thus, by combining the result of an antigen detection test and the CSBSS with a lower cut-off value, we can obtain higher overall sensitivity and specificity of screening without increasing the turn-around time or utilizing more healthcare resources. The rapid antigen tests combined with the use of CSBSS can give us a future direction to improve the sensitivity of the point of care test systems, which needs to be validated by further studies. This approach of combination of this score with rapid antigen tests may come up with promising results in near future.

The major strength of our study was that all the participants were tested with RT-PCR which is the gold standard for the diagnosis of SARS CoV-2 infection. The development of the scoring system was completely based on the RT-PCR results, which gives a high strength to the study design. The sample size was adequate enabling the scoring system to be replicable in different scenarios.

There are a few limitations in this study. Firstly, it was performed in a single outpatient clinic that serves our hospital staffs, healthcare workers, and their families. As the participants of this study are at a higher risk of acquiring COVID-19 infection and the infection prevention practices in this population are different from the general population, the results of our study may not be a reflection of the community at large [15,16]. Furthermore, this newly developed scoring system has to be validated in other settings to ascertain its uniformity. Secondly, the result of the CSBSS will not change the quarantine guidelines for a test candidate as the presence or absence of the disease will be confirmed on the basis RT-PCR reports only. Lastly, the nasal and pharyngeal swab samples for RT-PCR were collected by different healthcare personnel. The influence of the swab collection technique on the result of RT-PCR can not be ruled out.

## Conclusions

COVID-19 pandemic is not yet over in spite of the promise and expectation brought into our minds by the initiation of the vaccination programme. Infection prevention practices in the general population and early detection of cases still play a major role in mitigating the spread of the virus. We have developed a diagnostic scoring system using clinical symptoms to determine the probability of a person being infected with this virus. This simple and valid tool can be used for the risk stratification of the suspects before their RT-PCR reports are available. The scoring system can be utilized in areas where turn-around time for the RT-PCR is still longer; mostly in resource-limited settings and also in regions where the healthcare system is exhausted by the overwhelming number of patients. The final score can be calculated from the formula we have derived from a development dataset comprising 70% of patients and this scoring system has been validated in a validation dataset comprising the remaining 30% of the patients who attended the COVID-19 screening clinic in our hospital. Five clinical symptoms viz. loss of smell, fever >100°F, headache, cough and myalgia were statistically associated with the RT-PCR results. The scoring formula has been developed using these five clinical symptoms; thus making the system easy and convenient to be used as a point-of-care assessment tool in the diagnostic evaluation of COVID-19 suspects during this ongoing pandemic. The major limitation of this study was that it was performed in a single centre and the study population was not representative of the community. Differences in the swab collection techniques among different healthcare personnel might have influenced the results of the RT-PCR tests. Furthermore, the quarantine strategies for suspected individuals would not change on the basis of the score.
